# Providers' Shift to Telerehabilitation at the U.S. Veterans Health Administration During COVID-19: Practical Applications

**DOI:** 10.3389/fpubh.2022.831762

**Published:** 2022-03-04

**Authors:** Consuelo M. Kreider, Jennifer Hale-Gallardo, John C. Kramer, Sharon Mburu, Mackenzi R. Slamka, Kimberly E. Findley, Keith J. Myers, Sergio Romero

**Affiliations:** ^1^Department of Occupational Therapy, University of Florida, Gainesville, FL, United States; ^2^Veterans Rural Health Resource Center-Salt Lake City (VRHRC-SLC), Office of Rural Health, Veterans Health Administration, Salt Lake City, UT, United States; ^3^Department of Veterans Affairs, North Florida/South Georgia Veterans Health System, Research Service, Gainesville, FL, United States; ^4^Veterans Rural Health Resource Center-Gainesville (VRHRC-GNV), Office of Rural Health, Veterans Health Administration, Gainesville, FL, United States

**Keywords:** Veterans, qualitative evaluation, health care delivery, health care providers, access to healthcare, telemedicine

## Abstract

Telerehabilitation provides Veteran patients with necessary rehabilitation treatment. It enhances care continuity and reduces travel time for Veterans who face long distances to receive care at a Veterans Health Administration (VHA) medical facility. The onset of the COVID-19 pandemic necessitated a sudden shift to telehealth–including telerehabilitation, where a paucity of data-driven guidelines exist that are specific to the practicalities entailed in telerehabilitation implementation. This paper explicates gains in practical knowledge for implementing telerehabilitation that were accelerated during the rapid shift of VHA healthcare from out-patient rehabilitation services to telerehabilitation during the COVID-19 pandemic. Group and individual interviews with 12 VHA rehabilitation providers were conducted to examine, in-depth, the providers' implementation of telerehabilitation. Thematic analysis yielded nine themes: (i) Willingness to Give Telerehabilitation a Chance: A Key Ingredient; (ii) Creativity and Adaptability: Critical Attributes for Telerehabilitation Providers; (iii) Adapting Assessments; (iv) Adapting Interventions; (v) Role and Workflow Adaptations; (vi) Appraising for Self the Feasibility of the Telerehabilitation Modality; (vii) Availability of Informal, In-Person Support Improves Feasibility of Telerehabilitation; (viii) Shifts in the Expectations by the Patients and by the Provider; and (ix) Benefit and Anticipated Future of Telerehabilitation. This paper contributes an in-depth understanding of clinical reasoning considerations, supportive strategies, and practical approaches for engaging Veterans in telerehabilitation.

## Introduction

The United States (US) Veterans Health Administration's (VHA) has a mission to provide timely, high-quality care and services to all Veterans enrolled in the Department of Veterans Affairs (VA) healthcare. The VA operates the US' largest integrated healthcare system providing health care services and new programs to meet the changing and diverse medical, health, and quality of life needs of more than nine million enrolled Veterans (1, 2). VA research programs lead health care discovery and innovations to increase Veterans' access to quality care; these discoveries and innovations cut across other healthcare systems and benefit society (2, 3).

Chronic disability is highly prevalent in the United States with ~61 million adults reporting a disability in 2016 (4). Approximately 18 million Veterans in the US with 29.6% of Veterans ages 21–64 report a disability (5, 6). Rehabilitation is a key service within the VHA to help Veterans regain optimal function after impairment, maximize independence, and improve quality of life. Access to rehabilitation is especially challenging for Veterans with disabilities in rural communities, as well as for other patient populations who may not be able to attend in-person sessions for reasons such as distance, transportation, financial resources, or mobility challenges (7–12). Rehabilitation treatments often require several sessions, which can be challenging for maintaining continuity of care when travel to rehabilitation appointments is needed. Access to timely and quality care, especially for those living in rural communities, is a social determinant of health that is a priority for the US VHA (13–15). This priority is evidenced in the VHA Office of Rural Health's pre-COVID investments in the Telerehabilitation Enterprise-Wide Initiative (TR-EWI) (16) and the Rural Veterans Telerehabilitation Initiative (RVTRI) (17).

With the 2020 pandemic-related social distancing protocols for all healthcare systems in the US, the VHA leveraged its previous investments and experience in expanding telerehabilitation (i.e., TR-EWI and RVTRI) in service of the VA's system-wide adoption of virtual care. These initiatives are part of the VHA's commitment to enhance, through telehealth technologies, foundational services like rehabilitation–an area in which the VA health system has historically excelled. Telerehabilitation at the VHA includes Physical Therapy, Occupational Therapy, Speech Therapy, Supported Employment, Creative Arts, Recreational Therapy, Kinesiotherapy, Assistive Technology, Amputation Care, Polytrauma and Traumatic Brain Injury Care, and others (16, 18–22).

Telerehabilitation uses telehealth technologies to diagnose, evaluate, and manage health care for patients with physical, cognitive, and/or psychosocial impairment and disability (23). Telerehabilitation is a promising delivery method for Veterans with a variety of health conditions including stroke (24), multiple sclerosis (25), spinal cord injuries (26), amputations (27), and other conditions who could benefit from rehabilitation services (17, 28). Telerehabilitation has been used to facilitate the exchange of best practices between specialists who can remotely share their expertise and treatment alternatives for diseases such as cancer, heart attack, or stroke (16).

Telerehabilitation has been shown to improve access to care, augment clinical coordination and care continuity, and reduce unnecessary travel for Veterans (17). Within the VHA, telehealth technologies are used by rehabilitation specialists who are located at medical centers to connect with Veterans located at rural VHA Community Based Outpatient Clinics (CBOCs), at their home, or at non-VHA health facilities within the Veteran's community. In doing so, providers can evaluate, monitor, and observe patient participation in daily activities, community life skills, work, leisure, and social engagement (29, 30).

Beginning in 2020, the coronavirus pandemic (COVID-19) prompted a sudden change in health care that resulted in the rapid scale-up and implementation of telehealth (31–33). Despite this trend in health care, for patients requiring rehabilitation, there was an overall slower scaling-up to remote rehabilitation care related to the nature of rehabilitation activities (34). COVID-19, in combination with rehabilitation's protracted scale-up to tele-delivery of care, has brought to the forefront an imperative for rehabilitation clinicians and researchers to better understand the practicalities in how to deliver telerehabilitation services; and to do so in ways that are safe for patients and provides the quality that meets patients' and clinicians' expectations. The need for practical guidance in implementing telerehabilitation is evidenced by the breadth of anecdotal “how-to” guidance available through online sources. Within online rehabilitation communities of practice, practical information can be found on blogs and webpages offering resources, advice, and strategies for addressing discipline-specific considerations for telerehabilitation [e.g., (35–37)]. Published guidance, such as practice guidelines issued by professional associations [e.g., (38–40)] remains primarily broad. A gap exists in the research literature regarding understanding of the real-world practice of telerehabilitation that are gained through systematic investigation of practitioners' experiences.

We examined implementation of telerehabilitation within the VA healthcare system during COVID-19 and describe VHA TR-EWI rehabilitation providers' experiences of and practical strategies for implementing telerehabilitation - whose uptake was accelerated in response to the COVID-19 pandemic. The purpose of this in-depth examination was to identify key considerations, supports, and practical strategies for engaging patients in rehabilitation services *via* telehealth technologies for VHA rehabilitation providers.

## Methods

A qualitative approach was used to examine telerehabilitation implementation. Group and individual interviews, which lasted 45–60 min, were conducted with VHA rehabilitation providers (e.g., physical therapists, occupational therapists, speech/language pathologists, psychologists). Interviews were part of the 2020 evaluation of the VHA Telerehabilitation Enterprise-Wide Initiative (TR-EWI), which was deemed a quality improvement project by the VHA Physical Medicine and Rehabilitation Program Office (PM&R PO) in accordance with VA guidance, ORD Program Guide 1200.21, *VA Operations Activities that May Constitute Research*. The TR-EWI evaluation team is comprised of rehabilitation and health researchers with expertise in quantitative and qualitative research methods. The 2020 TREWI evaluation included an in-depth examination of VHA telerehabilitation implementation from the perspective of VA providers. This analysis focused on practical insights and lessons learned from the providers.

### Setting and Participants

In fulfilling the VHA's core mission of providing timely, high-quality health care to all enrolled Veterans, the VHA Office of Rural Health sponsored the 2017 national roll-out of the TR-EWI program. At its onset, TR-EWI employed a Hub-and-Spoke model whereby rehabilitation expertise is located at a Hub site and delivered *via* technology to Spoke sites that do not have in-house rehabilitation specializations.

TR-EWI program managers from each Hub site were asked to identify TR-EWI providers within their TR-EWI network (i.e., Hub or Spoke site). TR-EWI providers were clinical rehabilitation providers receiving any portion of their payroll support from the TR-EWI program or working within the TR-EWI framework but not supported by TR-EWI funds. Providers who participated in this qualitative inquiry were involved in TR-EWI during the 2019–2020 program year.

Program managers identified 36 eligible providers, 12 (33%) of whom participated in either a group or individual interview as based on scheduling availability. Two four-person group interviews (*n* = 8) and four individual interviews were conducted. Providers from each of the four TR-EWI Hub sites or the Hub site's affiliated Spoke site were represented, thus ensuring perspectives from across the VHA (Table 1). Data were collected in the second half of 2020, which was amidst the US national shutdown in response to the pandemic.

**Table 1:** Interviewee characteristics.

**Interview type**	**Clinical discipline (*n*)**	**United States geographic region**
Group	Clinical Psychologist (*n* = 1) Occupational Therapy (*n =* 2) Telehealth Medical Service Assistant (*n* = 1)	Mid-Atlantic Pacific Northwest South-Central
Group	Clinical Psychologist (*n* = 2) Occupational Therapy (*n = 1*) Physical Therapy (*n = 1*)	Mid-Atlantic Mid-West Pacific Northwest South-Central
Individual	Occupational Therapy (*n = 1*)	Mid-Atlantic
Individual	Physical Therapy (*n = 1*)	Mid-Atlantic
Individual	Speech Therapy (*n = 1*)	Mid-Atlantic
Individual	Occupational Therapy (*n = 1*)	Pacific Northwest

### Data Collection and Analysis

The TR-EWI evaluation team developed and interview guide and piloted it with a VHA clinical rehabilitation manager who is a physical therapist. The open-ended questions and probes developed for the interviews were used as a guide to ensure that all domains of interest were asked about. Consistent with the iterative nature of qualitative methodology, we anticipated that respondents could bring up additional salient topics regarding experiences serving Veterans under the TR-EWI program. Thus, modifications were made to the initial guide, with the final questions detailed in Table 2, whereby the respondent driven topics were asked about in subsequent group and individual interviews. This dynamic data collection strategy allowed for the pursuit of salient concepts generated by the providers, which contributes to the robustness of themes identified (41).

**Table 2:** Interview guide.

**Question number**	**Questions and probes**
1	Based on your understanding and experiences, how would describe TR-EWI's goals to others?
2	Thinking back to prior to COVID, then comparing to how things were at the beginning of COVID, and now that we're in the midst of COVID, in what ways has the TREWI program changed since you have been involved with TREWI or since you first heard of TREWI? [Probe]: What about your TREWI experiences with regards to extending rehab to rural Veterans? In what ways have you observed or experienced this? In what ways has this changed?
3	How would you describe telerehab's impact on your practice, and on VA rehabilitation practice, in general, prior to and after COVID? [Probe] What has been the impact of telerehab on your workload? Pre and post COVID? [Probe]: How did you determine (or what criteria did you use to determine) whether or not a patient would receive telerehabilitation or in-person rehab? [Probe]: What about impacts of TR-EWI or telerehab on your patients? Are they receiving different types or different focus of their rehab? Have there been any new services that you or others at your facility are able to provide because of the availability of tele-technologies (e.g., short follow-up appointments; telerehabilitation provider to vendor in the Veteran's community)? [Probe]: What sorts of compromises and or gains have been made in using telerehab? [Probe]: What are your thoughts and experiences regarding community care consults for PT? [Probe]: What are your experiences in integrating or interacting with your facility's primary care team?
4	Now that you've experienced telerehab prior to and during COVID, how do you perceive that rehab practices will be impacted in going forward? [Probe]: What things are you experiencing, related to post COVID telerehab, that you feel are worthy of sustaining or even expanding? [Probe]: What do you believe it takes to get a provider established in telerehab? What types of supports are needed?
5	From your experiences with tele – what can be improved to make your practice better? What is the best way to do your tele practice?

Group and individual interviews were conducted and analyzed for themes by members of the TR-EWI evaluation team with expertise in qualitative methods (CK, JHG, JK) and clinical expertise (CK, KF, SM). Following the thematic analysis, data were analyzed for content specific to practical strategies, considerations, and supports that had applicability across the rehabilitation disciplines represented in the sample. Emergent conceptualizations were regularly discussed with the entire evaluation team and verified by team members with VHA content expertise (KF, KM, SR). Group and individual interviews were conducted using video conferencing software (i.e., Zoom, Microsoft Teams) with use of video, chat, transcription, and recording functions agreed to by participants and used in all interviews. Transcripts were verified from the video recordings immediately after each group and individual interview and used for analysis.

## Results

The sudden interruption of in-person face-to-face health care delivery caused by the pandemic required a rapid shift in health care delivery. During the shift of VHA healthcare from out-patient rehabilitation services to telerehabilitation, gains in practical knowledge for implementing telerehabilitation were accelerated. Providers described a wide range of practical strategies and considerations for addressing challenges encountered in implementing telerehabilitation–strategies that helped to improve patients' experiences and their own workflow efficiency. These practical strategies and considerations were used in combination with administrative supports that together helped to facilitate the implementation of telerehabilitation sessions and enhance the rehabilitation process (Table 3).

**Table 3:** Strategies and supports used for implementing telerehabilitation sessions.

**Workflow process**	**Considerations and strategies**	**Corresponding themes**
During Chart Review and Scheduling	• Determine salient patient factors regarding appropriateness for telerehabilitation, considering factors such as falls risk, mental health status, caregiver availability, potential equipment needs.	1. Appraising for Self the Feasibility of the Telerehabilitation Modality 2. Availability of Informal, In-Person Support Improves Feasibility of Telerehabilitation
Setting up/Preparation	• Spend time ahead of sessions, if needed, to ensure clinician's skill with the telehealth technologies, including use of secure messaging features. • Create personalized checklists to use as self-prompts to prepare for sessions. • Create templates to guide session planning, observations, and documentation. • Develop written instructions that can be quickly adapted and disseminated to the patient. • Prepare back-up plans for communicating with the patient in the event of technical challenges while connecting *via* the primary technology application. • Consider and be prepared to support patients' (1) feelings of anxiety regarding not knowing what to expect with telerehabilitation, and (2) challenges around telerehabilitation technology use.	1. Willingness to Give the Telerehabilitation a Chance: A Key Ingredient 2. Role and Workflow Adaptations
Assessment and Intervention Planning	• Assessment: Make plans for substituting roughly equivalent home tasks or observable movements or actions for standardized objective measurement tools when needed. When using standardized measurement tools, consider patients' ability to follow instructions and adequately self-report. • Intervention: Be creative in intervention planning; devise functional intervention activities based on patient's environment, that appeal to patient, and maximize patient engagement. • Be ready to provide written materials and instructions *via* postal service mail and/or digitally before, during, or immediately following the session. • Coordinate with family members or caregivers so that they can participate in sessions, as appropriate.	1. Creativity and Adaptability: Critical Attributes for Telerehabilitation Providers 2. Role and Workflow Adaptations
During the Session	• Spend time building a therapeutic relationship with the patient virtually to establish rapport. • Support and guide the patient during technical challenges. • Continually engage patients during the session. • Focus and adapt the patient's session within their own context (i.e., unique home environment) and be prepared to use the patient's readily available household items. • Promote the shift toward self-management for Veterans with chronic conditions (e.g., working with the patient to allow them to actively participate and sometimes assist with problem-solving for how to adjust or contextualize assessments and interventions). • Ask family members to help with technology, equipment, and/or safety during sessions while constantly monitoring the patient, family members, and environment for safety concerns during the session. • Consider the patient's receptive communication skills and cognitive capacity for how it may impact the telerehabilitation session. • Provide ample educational materials, guidance, and demonstrations during the session.	1. Creativity and Adaptability: Critical Attributes for Telerehabilitation Providers 2. Adapting Assessments 3. Adapting Interventions 4. Role and Workflow Adaptations 5. Appraising for Self the Feasibility of the Telerehabilitation Modality 6. Availability of Informal, In-Person Support Improves Feasibility of Telerehabilitation 7. Shifts in the Expectations by the Patients and by the Provider
Administrative Supports	• Allocate clinicians extra rooms and quiet spaces to conduct telerehabilitation visits. • Allocate computers and accessories, such as earphones, to ensure privacy of the patient. • Seek assistance from rehabilitation technicians or technology personnel for conducting test calls (i.e., virtual visits) with patients and caregivers prior to the initial scheduled telerehabilitation visit.	1. Willingness to Give Telerehabilitation a Chance: A Key Ingredient

### Willingness to Give Telerehabilitation a Chance: A Key Ingredient

The rapid pivot to telerehabilitation within the VHA required adaptability and creativity on the part of providers. It also required administrative support by medical facility leadership and rehabilitation managers, as well as a willingness to accept a new form of rehabilitation service delivery from the Veterans themselves. Across multiple levels, a willingness to try telerehabilitation, adapt as needed, and persist with use of available telehealth technologies was a key ingredient for successfully shifting to telerehabilitation services.

#### Administrative Supports and Resources

VHA's rapid shift to telerehabilitation pandemic-related shutdown was supported by existing TR-EWI infrastructure and expertise. This infrastructure included a three-year-old team of program managers and rehabilitation clinicians with experience delivering telerehabilitation assessments and interventions to rural Veterans. This team also had experience in developing and delivering training to other rehabilitation providers in the implementation of telerehabilitation through TR-EWI.

Additional sources of support for VHA providers' transition to telerehabilitation came from medical facility leadership and rehabilitation management, information technology (IT) technicians, and other care providers engaged in the provision of telehealth services. Administrative support from facility leadership and rehabilitation management included the availing of extra rooms and quiet spaces – as well as the computers and accessories needed to ensure patients' privacy during telerehabilitation visits. As described,

*“[We've] set up all of our work workstation on wheels…All of them have…webcams… their own set of headsets with mics on them so that we can…limit the amount of noise that's going in and out of [the clinic].”* [S002]

IT personnel at some facilities assisted by conducting test calls with the patient and/or caregiver prior to the scheduled telerehabilitation sessions. This helped prepare the Veteran to navigate the available technology. Health care providers from outside of rehabilitation also leveraged their experience and shared knowledge gained during their own telehealth sessions with other VHA providers, including those in rehabilitation.

#### Patients' Willingness

Prior to the pandemic, both providers and patients had reservations about use of the telehealth modality for rehabilitation.

*“I think that there was a lot of kind of anticipatory anxiety about trying [telerehabilitation] all at the beginning. And now that everybody's just been forced to do it; then both the providers and the patients are going to be a lot more comfortable with it…I think we'll have more providers…willing to do telehealth on a regular or semi-regular basis. And I think that we're going to have patients asking for it.”* [S006]

Initially, patients' acclimation to telehealth technologies for rehabilitation visits was supported by the adoption of telehealth by medical primary care providers.

*“Since COVID, I have found that for my new patients at least, I'm usually not their first exposure to telehealth. You know, they've already done a primary care appointment or PT appointment or, you know, something like that so they're already familiar with it.”* [S006]

Patients' previous exposure to telehealth from other types of health providers helped Veterans anticipate what to expect during telerehabilitation sessions.

The societal shift to extensive use of remote technologies brought about by the pandemic was perceived as a helpful primer for Veterans' acceptance of telerehabilitation.

*“I think the fact that a lot of that physicians in the outside world are also now using telehealth [has]…helped…to bring patients into the fold [for telerehabilitation].”* [S001]

### Creativity and Adaptability: Critical Attributes for Telerehabilitation Providers

When providing telerehabilitation, providers spoke of new clinical challenges experienced when first working in the remote rehabilitation environment – challenges that required creativity and adaptability of the provider to overcome. As shared,

*“Some things you can't do virtually, but other things can be adapted; it just takes thinking outside the box a lot of the time… recently I've seen a huge, huge growth in how people are looking at things. We're coming at it from a different paradigm [now, since the pandemic] for the most part, and, and the big, big difficulty [before the pandemic] was getting people [i.e., other providers] on board initially - early on with TREWI, to look at it from that different perspective.”* [S009]

Regardless of initial challenges, which for some included resistance to change, many providers felt that the shift to telerehabilitation was inevitable, and that the COVID-19 pandemic only accentuated this inevitability despite perceived resistance to change by other rehabilitation clinicians.

*“…Not to make light of the situation, but everyone said the same thing about the microwave. No one wanted to use a microwave 35 years ago. But here we are within a microwave society, you know, so in saying that it's the same thing with tele rehab. This is here and it's not going anywhere. So, we as therapists, regardless of our occupational background in healthcare, we have to find a way to adapt.”* [S003]

Providers also spoke of initial challenges in developing the therapeutic relationship with their patients *via* telehealth technologies, requiring adaptation and learning around how to sustain the patient-provider relationship at a distance.

*“I think I've adapted to learning how to have a relationship with your patient over the video vs. a face to face [in person visit]… I think learning how to do that via … a video screen vs. face to face … I thought that would be a compromise, but I think I've adapted, so I think I wouldn't consider it to be one now…”* [S010]

Providers discussed the importance of continually engaging the patient during the telerehabilitation visit. They spoke of working to keep patients engaged in the telerehabilitation process, and thus, motivated to persist should frustrations, such as technical challenges, occur during the session. In doing so, providers shared how they leveraged the technology as one means of engaging patients in the session.

*“…I really enjoyed the share the screen feature - I use that a lot. I can pull up the exercises on my other screen so they can see that. And I usually just mail them a hard copy as well. But just having that availability. They really like that. Or, if I'm showing them how to [use] a specific brace, [that] they're getting ready to receive, [then] I'll show them [what to expect], I can Google a picture and share the screen with them.”* [S009]

Several also emphasized the importance of incorporating a focus on the Veterans' functioning within the context of the Veteran's unique home environment and a focus on what is practical and appropriate to the telerehabilitation encounter. As one participant stated, “*The telerehab has to be done more in a functional standpoint vs. the clinical.”* [S002]

Often providers were able to use items within the patient's home environment – during both assessments and interventions, which served to make activities selected within the session more obviously applicable to the patient as compared to therapeutic activities engaged in within the rehabilitation clinic. As described by another provider,

*“…Instead of, ‘okay, [in clinic] I have my rubber bands… my TheraBands, and I can do your exercises, I can do 15.’ Now [during the telerehabilitation sessions] it's like, ‘Okay. Great. Now let's see if we can do that [motion] while you're lifting the laundry, the laundry basket.’ So, it makes it so it's more…realistic - and it is truly functional. It makes sense to their day-to-day.”* [S012]

Some described leveraging telehealth technologies to expand their group rehabilitation offerings (e.g., Parkinson's vocalization, meditative movement groups), whereby they incorporate Veterans virtually into existing in-person groups or hold the entire group session remotely. As shared,

*“I am going to be starting a Tai Chi group remotely with select Veterans and so I'm already going to see somebody from Alaska, who's joining my group and then I have one person who's in [the contiguous US] who, so neither one of them would have been able to participate in my group anyway if it was face-to-face, so it's providing them with an opportunity to receive services that would not be available to them…it allows them access to care.”* [S010]

### Adapting Assessments

Providers had to change approach when conducting clinical assessments *via* telerehabilitation. Several spoke of being initially challenged to find creative and adaptive solutions needed to bridge the shift from traditional in-person or hands-on clinical assessment methods and measurement tools. Many shared how they overcame such challenges by shifting the assessment to a more functional focus with careful clinical observation.

*“I use…my observation skills and looking for movement patterns to… solidify what I would do for that patient…So, I use it [assessment] more like a functional way to observe their capabilities, rather than relying on my hands-on approach [when assessing]…”* [S012]

For instance, providers substituted familiar objective measurement tools from the clinic with what they considered roughly equivalent home tasks or observable movements to gauge the measurement.

*“…I could ask them to lift something that I have an idea of what the weight of it is, I could say, pull out a 16 ounce can from your cupboard. Can you pick that up from your cupboard and take it down to the deck? So, I, in a sense – in a round-about way - I can potentially do a manual muscle test. I can see, but it's more from a functional standpoint, versus…purely trying to get a specific muscle grade or range of motion…”* [S002]

In other instances, providers needed to modify existing assessments to capture the information most relevant to the patient and the patient's specific environment.

One spoke of the multiple steps it sometimes took to walk some patients through to prepare them for engaging with the video-based telerehabilitation software application:

*“…I find that sometimes when I do a telephone visit and they get to know me, then they're more [open to telerehabilitation], and if I try to help simplify the VVC [i.e., VHA's video-based telerehabilitation software application] experience, they might be less intimidated. Sometimes if I do the test call vs. somebody else [such as the scheduling assistant] they might feel more comfortable and not so overwhelmed.”* [S010]

Another spoke of the importance of patient communication skills during the remote clinical evaluation process:

*“I think a lot of it is their [i.e., the patient's] ability to communicate effectively. I won't have my hands there to position them accordingly. So, they need to be able to really understand and follow instructions one way or the other…I need to make sure that they really understand that I'm going to have them move around, but I need you to be able to follow instructions accordingly and not move on your own before we go over the, the process so that I can make sure they don't have falls or I don't miss anything for that matter.”* [S012]

Providers also had to consider assessment tools' ease of use. This was especially true in instances where the patient was asked to use an assessment tool independently, such as self-report measures. In instances where the patient's clinical condition impacted cognitive capacity, providers needed to consider abilities to remotely follow assessment-related instructions and adequately self-report.

When using self-report instruments, some providers chose to mail *via* postal service materials or questionnaires in advance of the scheduled session. These providers emphasized the importance of minimizing the cognitive and technical load of downloading, in real-time, a document during a session. Ensuring that patients had written materials ahead of the session freed up valuable session time and allowed more time to review with patients the documents or questionnaire items, thus ensuring that critical assessment questions are answered.

### Adapting Interventions

Providers spoke of needing to adapt planned intervention activities based on specifics of the patient's home environment. For example, providers spoke of needing to account for the patient's preferred seating locations during scheduled treatment sessions. This consideration was especially cogent for patients whose intervention included aspects of physical rehabilitation or functional movements, and who expressed preference for engaging in the telerehabilitation from a seating arrangement that is not optimal for the planned treatment activity. One physical therapist spoke of being especially challenged in developing mobility-related therapeutic activities for the patient to engage in while primarily seated on a preferred plush couch.

Providers spoke of needing to devise activities that appealed to the patient, created optimal instances for engagement in the prescribed therapeutic activities, and kept patient safety in mind.

*“What are some means of keeping it exciting so you want to continue to do therapy, just like we are in the clinic? But also a way that I feel comfortable as a provider that you're safe that you're, you know, you're compliant. And so I think being creative [is key]…”* [S009]

Overall, incorporating the home environment into planned treatment sessions required a level of creativity that several reported enjoying as a new type of challenge that contributed to professional growth. Providers cited simple adaptations such as having their patients engage in overhead reaching exercises *via* functional activities such as putting grocery items away on high shelves, and having patients use a gallon of milk and cans of green beans in place of the traditional dumbbells that are typically available in rehabilitation clinics.

*“…I think the whole thing [i.e., telerehabilitation assessment and intervention] is just creative…[for example] the whole…process, I can't touch you, so how can I measure your strength or how can I assess your balance? So just trying to find some objective measures, how to research a couple objective measures that were easy to do so that they can do on their own and could still give me a good idea of their progress throughout their plan of care.”* [S009]

Another provider described really appreciating being able to create intervention plans that were very client-focused and meaningful to the client.

*“…I love being able to see them in their home environment. So, you're describing this chair that you can't get out of at home, but now I can see it. And we can work in that manner. So, I really enjoy it. I think that my patients really enjoy it.”* [S009]

### Role and Workflow Adaptations

Not unexpectantly, providers shared how their initial shift to telerehabilitation required not only additional time for learning the technology, but also for figuring out which types of assessment and intervention approaches could work *via* telerehabilitation. Providers also had to determine what types of patient education materials and tools would be useful within their remote interventions. For several, preparing for sessions entailed preparations that were different in nature than preparing for a patient who would be seen in person. For example, when preparing for telerehabilitation, providers had to identify therapeutic activities or home exercise guides that the patient could engage with independently. Once located, providers needed to ensure that all therapeutic activity guides or visual examples were in digital format and easily accessible within the session. In describing what it takes to prepare for a telerehabilitation session:

*“There are a lot more paths to check, mainly because most of the Veterans are not familiar with the VVC [i.e. VHA telerehabilitation software application] and in the meantime I have to put on my IT hat vs. my [clinician] hat, you know…Meaning calling them before the actual appointment to make sure that they are actually safe to do a VVC [i.e., video] appointment, makes it so that time can get away from me trying to make it happen and make it billable at the same time. So, there are admin work in between that needs to happen, but I'm finding that as I go along, I'm more efficient now than I was in March [i.e., March 2020 at the beginning of the US pandemic-related lockdowns].”* [S012]

With the sudden rise in the sheer number of telerehabilitation sessions that came with the pandemic, providers were especially pressed to find ways to streamline workflow, which at times entailed a willingness to engage in activities typically considered outside rehabilitation – specifically, technology.

#### Providers' Expanded Role

Providers spoke of the critical nature of working technology – of having to take the time to ensure that the technology was always working correctly. This was seen as a basic but fundamental piece of preparing for the telerehabilitation session and thus, periodically taking the time to have a technology focus was seen as something that they could do to ensure a better patient experience. Some spoke of feeling the need to build extra time into their daily schedules for potentially assisting patients with things like technical guidance.

Some providers reported having to put on their ‘*IT hats*’ and screen patients for technical readiness to engage in telerehabilitation. Several spoke of the importance of being technologically proficient enough to guide patients through unanticipated technical challenges – or at least create a plan to complete the telerehabilitation session by telephone if needed. Unfortunately, such occurrences, while necessary for some patients, eroded available session time. In other cases where more extensive troubleshooting was required, providers had to reschedule the session.

#### Strategies for Improving Workflow and Efficiency

Providers spoke of using other technology tools to counter workflow inefficiency. One provider spoke of the helpfulness of using electronic secure messaging features when needing to digitally transfer documents or to share necessary information, such as a website with helpful instruction for patients. Use of the secure messaging feature eliminated providers' need to spend additional time preparing materials for the session or following up with an email or mail *via* postal service containing written versions of the needed information.

Providers also spoke of the helpfulness in being able to use readily available digital tools during patient education. While patient education could easily be delivered during telerehabilitation sessions, multiple providers spoke of the importance of making written instructions available to patients. Doing so often entailed providing the materials either ahead of remote sessions or *via* screen sharing tools during the session. For patients whose technology literacy was inadequate for accessing a digital version while also accessing the telehealth application, this meant providing the written materials *via* postal service mail or relying on a caregiver to assist with downloading and printing or navigating the technologies to enable digital access during sessions.

In working to improve efficiency and facilitate the success of the telerehabilitation sessions, some created checklists. Checklists were used to prepare for the telerehabilitation and to facilitate follow-up after sessions. Providers described checklists that they used as self-prompts that directed them to adequately prepare - for instance: (1) have open - in advance - any digital files, such as webpages or pictures, that may be used in the treatment session; (2) have ready written instructions for the patient; and, (3) in the case of patients who need home exercises programs, locate digital files of exercise or activity instructions that can be easily modified or customized based on what is available in the patient's home. Providers noted that often they needed to tailor written instructions according to the needs of the patient. As such, many developed strategies for extemporaneously furnishing patients with custom-crafted instructions during or immediately after the assessment or treatment session.

Providers spoke of shifts in what they documented during and about the rehabilitation sessions. They reported creating templates to guide assessment and documentation during the sessions. Templates contained detailed prompts that guided to note qualitative aspects of patients' home or environment. This involved noting details such as the position of a table or chair used in the session, and specifically how the in-home items were used.

*“…I'll document, like, what surface we're using in the house …so it kind of helps jog my memory… plan for next week…[I can say things like]'we're going to practice you getting up from your recliner because that's where you sit most of the time during the day'…”* [S009]

Detailed qualitative documentation was also valuable in helping providers quickly analyze and understand patients' challenges within the home. This level of detailed documentation was advantageous for planning personalized sessions.

*“…[I] try to document as much as I can. It helps me remember…guide their treatment and… [it] adds a little bit of a personal touch …I try to try to document it all so I don't forget it and it can help me … guide my plan of care and my treatment.”* [S009]

Detailed qualitative appraisal also enabled providers to plan for the presence, or lack, of caregivers to help with the rehabilitation process. Detailed documentation was also beneficial in capturing patients' severity and variations in capacity to maneuver within the home. One provider noted:

*“…with documentation…, everything is grossly observed, functional strength is viewed as this [level] based on x, y and z [as observed during the patient's performance in the home environment]. So, it's me really watching everything that they're doing [and documenting those details].”* [S012]

As such, documentation was key in guiding the provider when making clinical decisions.

#### Appraising for Self the Feasibility of the Telerehabilitation Modality

Providers spoke of needing to be able to determine the goodness of fit between the telerehabilitation modality, their own ability to adapt preferred therapeutic strategies, and the individual patients. Appraising patients' goodness of fit required estimating which patients would likely accept the modality, be resilient to overcoming unexpected challenges, had the physical and mental abilities to engage in the session, and whose home environment was conducive to supporting successful telerehabilitation. Also critical in shaping providers' appraisal of telerehabilitation as a feasible modality within their practice, was the availability of patient support at home, such as family members or caregivers.

#### Considerations Around Therapeutic Approach

Providers also had to assess the type of treatments that could be feasibly provided remotely or adapted from in-clinic delivery. These appraisals needed to be made in consideration of each patient's condition or clinical presentation.

*“… we would look at… are they safe to do this?… are we putting ourselves at risk are we putting the patient at risk for anything? Are they appropriate to be seen?…will their medical diagnosis allow us to see them [via telerehabilitation]?”* [S002]

Even when sessions were deemed appropriate for telerehabilitation, a few spoke of instances when they still needed to work in-person with patients for some portion of the treatment plan.

*“I feel like swallowing is probably what I pay most attention to and making sure that I'm not compromising in an area because there's a safety risk with that… whereas in person, I could lay my hands on them [and] like make better recommendations. And sometimes… especially in the areas where internet's not great, I can't always see the Veteran - or I'm not seeing it in real time, because there is a lag. So I'm like, I don't know if I necessarily want to make this recommendation, just based on the information I was able to gather [remotely]. So in those situations…I tell the Veteran… I need you to come into the clinic because I need to actually see you a little bit better…to make a decision.”* [S011]

“*And one other thing that I'm thinking of now is in our field [of psychology]…[sometimes I have to give] feedback where there was potentially really bad news…I never really wanted to do [that] over telehealth…[I've] thought a lot about the ethics of doing that and how to manage [the situation]…if I'm telling somebody [really bad news about their condition or] cognitive decline…At times, [I've] brought people in in person [rather than tell them over telehealth].”* [S004]

Reasons for needing to intersperse periodic face-to-face visits varied and were often discipline specific. However, despite the occasional need to intersperse face-to-face visits into a telerehabilitation plan of care, all providers were enthusiastic about the strengths and opportunities that the telehealth modality offered and reported success with incorporating it into their care plans.

#### Considerations Around Patient Condition

Providers shared considerations about the impact of patients' mental status on decision-making around how to best use telerehabilitation. Some spoke about how, for those with mental health conditions, the ability to provide telerehabilitation could be advantageous for the patient, especially in cases where the Veteran's anxiety was exacerbated by having to come into the medical facility for appointments. One interaction with a patient was shared as follows:

*“He said, ‘You know what, I'm glad you guys have this [telehealth] because I usually get anxious when I come to the VA.’ So, having…this setup, you know, does allow him to interface and definitely make his appointments without having as much built-up tension, so to speak, you know, from his own diagnoses.”* [S003]

One provider, a clinical psychologist, spoke about important safety considerations for certain clinical populations, such as polytrauma patients, who are at high risk for suicide. This provider spoke about facing a dilemma in deciding how to safely work with these patients *via* telehealth, or if telehealth is even an appropriate option for these patients. As shared,

*“…We've spent a lot of time thinking about risk, mostly related to mental health risk… over telehealth. You know, you come for the intake in person, and then you can do follow up. We, the psychologists…would see folks when we thought it was clinically appropriate the first time over telehealth. But there were certain clinics- Polytrauma was one in particular, that we tried to see people in the CBOC [i.e., community-based outpatient clinic], because the rate of high-risk suicide issues coming up in that population…is really, really high… [We have to weigh out,] ‘Is this somebody who's not going to be seen at all?’ And then [we have to ask] ‘What are the ethical and risk implications of that, of not seeing somebody at all because they're too high [a COVID] risk and can't come in?’ Or, you know, are we comfortable managing various levels of suicide risk and disruptive behavior remotely?”* [S004]

For patients with mild functional impairments, adapting the treatment approaches and activities for telerehabilitation was more straightforward and entailed a heavier emphasis on patient education. However, for concerns regarding patient safety within the telerehabilitation visit, providers were more likely to choose in-person clinic visits.

*“…I try to keep a super open mind on people that are or are not appropriate [for telerehabilitation] … [If] you're having several falls a day… it's just not safe for you to be on your own at all, then I would say I'd rather see you in the clinic, just from a safety standpoint…”* [S009]

In the rapid pivot to telerehabilitation, the safety of the patient in relation to their environment and themselves (i.e., the patient's cognitive capacity), weighed heavily in providers' appraisal of telerehabilitation's feasibility. Clinicians needed to weigh considerations of any impairment associated with the Veteran's polytrauma, mental health status, and communication abilities.

### Availability of Informal, In-person Support Improves Feasibility of Telerehabilitation

One key aspect in estimating which patients would likely be successfully treated *via* telerehabilitation was the presence of, or ability to establish, a supportive network of others who can assist the patient in engaging in the remote rehabilitation process and help the patient meet the rehabilitation goals. Oftentimes, this supportive network included family members, but could also include other informal supporters, such as neighbors or church members (herein after, we use the terms “family” and “family member” to encompass the range of informal in-person supports available to patients).

Providers expressed that the presence of family members, and their help in managing sessions, served to enhance the telerehabilitation experience. In some cases, providers were able to avail themselves of family members to confirm or determine discrepancies in information that may have been relayed by the patient.

Patient families who were present and engaged during sessions played important roles in troubleshooting technical problems, assisting with safety concerns especially for Veterans with significant impairment, and assisting Veterans in reaching their goals, such as supporting follow-through with exercises recommended outside of the telerehabilitation session. The inclusion of family members was an added support that enhanced the rehabilitation process.

#### Technical Assistance

Family members helped troubleshoot technical problems before and during sessions. One of the ways that family members showed to be an important support was by managing technological aspects, such as opening the telerehabilitation platform/application for the patient. During sessions, family members could be helpful in holding the phone (camera) so that the therapist could better observe the patient. One provider shared:

*“…if they have a spouse or a caregiver available, [I can ask the patient], ‘Can they hold your phone for you so I can see how you're walking with your walker, and I can see how you're negotiating your house, how you're getting in and out of the bed or things like that’?”* [S009]

Another provider noted that patients' younger adult children were often intuitively helpful when assisting, such as dynamically adjusting the camera angle to give both the therapist and the patient the best lines of sight for rehabilitation activities.

Family members also helped with measuring items such as power mobility seating, which they measured under real-time instructions from the therapist. Family members were also important supports for providing real-time clarifications; this was especially important for patients whose condition involved difficulty speaking. Providers also spoke of the significance of having a family member in the patient's home when severe cognitive impairment or decline was present. Providers spoke of needing to communicate with family members to confirm what was being reported by patients with cognitive impairments, especially when what was reported is a potential safety risk.

*“I've called their family members after sessions, just to make sure; [its] like, ‘Hey, this is what we talked about, he said that he's cooking' and the family will say, ‘Oh, no, no, no, he doesn't cook’.”* [S011]

#### Assistance With Safety

Family members played a role in ensuring patients' safety during some telerehabilitation sessions. Providers reported that family members are especially helpful in sessions that require a hands-on approach. One provider spoke about issues related to managing medication:

*“I would say the swallowing people are the ones that I probably have the greatest concerns with - like even [compared to] the patients with dementia that I've seen. [When] there's a caregiver present…that I can talk about safety concerns or things to do in the home [its helpful] - I think that telehealth has helped address some of those safety issues that I probably would have a greater concern [about] if they were [seen] in person…[its] like [when] a Veteran tells me ‘I'm managing my own medications’. I say, ‘let me see’. And then [because its telerehabilitation into the patient's home] I [can see all their meds sitting in a sink and I'm like, ‘Oh, you're not really managing your medications. Let me get you a pill box and let's set that up together’.”* [S011]

Others spoke about the importance of having available family members during sessions that require patients to move around the home, thus reducing falls risk.

*“I'm being …creative in a manner…for fall risk [and]… [I can ask myself] ‘what's the safe way that we can work on strengthening balance training [that] incorporate the family?’ if they have a nice supportive family system.”* [S009]

#### Assistance With Medical/Rehabilitation Equipment

Setting up equipment, such as a walker, or ensuring that equipment is safely and properly set up is an area where family members can be particularly helpful.

*“Mostly everything I can do, I can do over telehealth. It just may require more assistance from [the] family depending on how severe they are. But the people that [have to] come in person are [the] people that need the equipment setup.”* [S011]

Family members also helped coordinate health care appointments and facilitate communication with other care providers.

### Shifts in the Expectations by the Patients and by the Provider

Providers noted the need for a shift in expectations by patients regarding patients' level of engagement and participation in the rehabilitation process when delivered *via* telerehabilitation. They reported that rural Veterans who had received referrals for rehabilitation prior to pandemic-related shutdowns often sought non-VHA rehabilitation care closer to their home rather than traveling to a VA Medical Facility. In response to shutdowns, Veterans with active rehabilitation referrals were contacted and offered telerehabilitation to ensure continuity of care. A few providers shared how some Veterans with chronic conditions received passive treatment modalities such as muscle massage or stretching, during their in-person community-based visits. For these patients, the telerehabilitation sessions required that they actively engage in therapeutic activities or exercises without the physical presence or hands-on assistance of the provider.

As such, telerehabilitation was perceived by providers to empower patients through greater emphasis on condition self-management and establishment of self-care behaviors and routines. In describing the process for helping the Veteran make the shift, one provider shared:

*“I'm not doing it for you. You have to show me that you're doing this, you have to show me that this is actually working, you have to show me that you can progress… And I think that is the big concept,…self-management,… which I think is the biggest change [with the shift to telerehabilitation].”* [S012]

The shift to the remote rehabilitation environment increased patients' responsibility in monitoring their own progress with prescribed therapeutic activities, as well as in providing feedback to the provider - especially feedback regarding aspects of the activity that may not be visible to the provider through the video camera.

Successful adaptation to this shift in expectations often hinged on effective interpersonal communication between the provider and the Veteran, and each of their abilities to clearly convey expectations and goings-on. Providers needed to deliver simple, concise, and direct messaging regarding expectations in the moment and in preparations for the next visit. Providers also needed to empower patients in the telerehabilitation process through provision of sufficient patient education. Providers reported that Veterans who were initially reluctant to engage in telerehabilitation often warmed to it with proper support about the modality and processes.

Overall, the general opinion was that Veterans – even ones who might at first feel uncertain or reluctant – could benefit from telerehabilitation, especially if the necessary restructuring and frontloading of time and expectations was managed by the provider:

*“… If you put more time up front, then you're likely to have a successful outcome…If they're feeling anxious, then they're less likely to…attempt to…open up. I've had that experience with where they felt that, and then if you talk to them through it…Many of them, often times feel like, ‘Oh, wow. It wasn't that hard’.”* [S010]

Providers also noted that reframing Veterans' options for rehabilitation care was important in helping patients understand the possibilities of telerehabilitation. Reframing also helped providers understand the Veteran's motivations for continuing community-based care rather than receiving rehabilitation care through the VA health system. As explained:

*“I think just kind of letting them know that [telerehabilitation is] an option that's available to them, so maybe we could keep them in the VA system instead of doing the community care… I don't blame them [for preferring rehabilitation care in their own community]… if it's two or three hours to go to a [VA facility] therapy appointment, I don't know if I'd be willing to drive that far every single time when I could go a little bit closer. But if I know that I could still be seen by those same [VA] providers virtually, you know that could open up some doors there as well.”* [S009]

Even when Veterans had a history of choosing local community care due to geographic convenience – such as when Veterans live in rural communities, that oftentimes they will choose to access VHA telerehabilitation once they understand how it works and what they can accomplish. In many cases, the telerehabilitation option can be even more convenient for the patient.

### Benefit and Anticipated Future of Telerehabilitation

Providers overwhelmingly reported that many, if not most, out-patients they serve can benefit from telerehabilitation.

*“And I think it's particularly pronounced for the folks that we work with, who largely have a number of disabilities that impact their ability to easily get into the VA. So, they might not be living necessarily in a rural environment, so to speak, but the challenges for getting even from … their home in downtown … to the VA are enormous.”* [S002]

“*…Any of the things that we used for the rural Veterans will work with anybody…that has the capability of using telehealth.”* [S004]

Several spoke of enhancements afforded by telerehabilitation sessions that took place at the patient's home. This enabled providers to customize interventions - as based on the patient's unique situation and home set-up, in ways that are not as easily afforded when seeing patients in the rehabilitation clinic.

*“I think it may also be a more efficient use of time - in terms of appointment time with our patients where we're not necessarily having to spend as much time linking a patient in and getting them set up for some things…[With telerehabilitation] we can better use their appointment time and gear it towards their specific needs, [especially] if we're seeing them [via telerehabilitation] in the home…”* [S011]

Telerehabilitation was also viewed as beneficial to the VA healthcare system.

*“I think especially for our [mental health] population, increasing access to care only benefits Veterans and…[VA healthcare] services. So yeah, I don't see this going away and it's a good option. I do think it'll probably be a mix of folks coming in [for face-to-face] and…virtual care.”* [S005]

A few participants spoke about positive impacts of telerehabilitation adoption for clinicians. Providers had to be creative and adapt interventions, which many enjoyed, and was viewed as an unexpected source of professional growth. Some spoke of how, during the pandemic-related shutdown, telerehabilitation enabled more providers to tele-work, potentially increasing more opportunities for hiring providers that can serve rural locations.

*“I think that the combination of doing telehealth and telework is going to give us an opportunity to bring in a lot more providers. So…that's going to improve access to care.”* [S006]

The VHA's widespread adoption of telerehabilitation was seen as helpful to recruitment of clinical providers. This was considered a cogent service-delivery strategy for enhancing Veterans' access to rehabilitation care.

Overall, regardless of challenges providers experienced during the rapid, wide-spread adoption of telerehabilitation, they were enthusiastic about telerehabilitation's future.

*“I know that sometimes it's hard to change and if you're used to practicing a certain way, sometimes it's difficult to modify that. But I think just the more that we use it, and COVID honestly…kind of forced us to use it a lot more than we probably would have…[now that I] have been using it…I'm just excited about it. I think it's just a whole other realm of, of interventions and therapies…I'm just excited that at the VA I'm able to kind of explore it. So hopefully it'll…just continue to grow.”* [S009]

“*I think this is going to be standard of care now here. I think it'll be incorporated into our practice. We'll do some hands-on and then transition the patient to telehealth. Or if they live too far away, it'll be [where] the Veterans will even be calling and asking, ‘Can a physical therapist do a telehealth visit with me, for my shoulder pain, on that lunch from 12 to 1?’… I think it's definitely going to be…a standard in our practice, a normal conversation and I think…that's what it's going to be – at least, I hope.”* [S007]

Clinicians easily envisioned continuing to incorporate telerehabilitation into their practice when pandemic-related restrictions to face-to-face visits are no longer needed.

## Discussion

The COVID-19 pandemic accelerated innovations within the VA healthcare system. Gains in practical knowledge for implementing telerehabilitation were advanced during the VHA's rapid shift from out-patient rehabilitation services to telerehabilitation in response to the pandemic. This paper reports key processes used by VHA rehabilitation providers from physical therapy, occupational therapy, speech-language pathology, and clinical rehabilitation psychology disciplines in implementing telerehabilitation. Findings contribute much needed data-driven telerehabilitation information (i.e., primary research findings) that can provide specific procedural or practical telerehabilitation guidance. Moreover, findings include information specific to movement considerations and safety considerations for patients with physical disabilities or impairments impacting cognitive functioning. A 2021 scoping review of telerehabilitation guidelines regarding patients with physical disabilities found only three studies providing specific provider guidance; however, the guidance was specific to use of the telerehabilitation technology (42). These researchers concluded a notable lack of movement-specific related clinical telerehabilitation guidance.

Providers weighed clinical considerations, identified necessary supports, and created practical strategies for delivering telerehabilitation. Telerehabilitation, when thoughtfully and creatively implemented, can provide increased opportunities for both patients and clinicians. Findings indicate potential enhancements to rehabilitation care plans and the rehabilitation care continuum that are brought about by telerehabilitation's incorporation into clinical practice. Findings extend understanding of the ways in which telehealth can be leveraged to improve patient experiences of their needed health and rehabilitation care beyond improved convenience, privacy, and patient comfort (43).

We found that outpatient rehabilitation activities are not typically thought of as easily converted to telerehabilitation due to the hands-on nature of VHA physical, occupational, and speech-language therapies. Telerehabilitation required providers to don two “hats” during remote sessions; that of clinician and that of technological support. As clinicians, telerehabilitation requires abilities for approaching evaluations and interventions with creativity, persistence, and in ways that foster rapport and engages patients remotely. This finding aligns with reports describing the ways in which telerehabilitation, in its constraints of no physical contact, requires a focus on different clinical skills than those typically used to physically guide patients during in-person sessions. Telerehabilitation entails increased reliance on subjective examination and, thus, enhanced interview skills, as well as abilities for systematic problem-solving (44). When patient movement is involved, sound understanding of movement patterns is also required (44).

While therapists' resistance to implementation of telerehabilitation may exist, clinicians, for the most part, can quickly become adept with the modality (44–46). We found that some clinicians even considered the creative and learning aspects of telerehabilitation a positive challenge that contributes to professional development. This examination yielded a broad range of clinical considerations and practical strategies used in implementing telerehabilitation in an efficient, safe, and functionally client-centered manner. Practical strategies identified address a gap in the empiric literature that can be useful in guiding VHA clinicians' decision-making when shifting practice to incorporate telehealth modalities with adult out-patient clients.

Shifts in how assessments were conducted and how interventions were structured in the telerehabilitation sessions placed greater responsibility on the patient or caregiver for active engagement in the rehabilitation process. Remote rehabilitation service delivery required empowering the patient to carry out therapeutic activities as guided by the clinician, rather than engaging as a more passive or physically-assisted recipient of the rehabilitation modality/activity. Barriers to physical therapy telerehabilitation include patients' negative perceptions of telerehabilitation related to the lack of manual contact during remote sessions (47). For some with longstanding rehabilitation needs, telerehabilitation required a shift toward greater self-management. Self-management refers to the active coping strategies and activities employed by patients with chronic conditions (48). findings suggest that telerehabilitation, by virtue of its remote, and thus hands-off nature, may be leveraged toward advancing patients' self-management skills and thus potentiating patients' maximum daily functioning. Future studies are warranted to examine the impact of telerehabilitation on patient self-management.

Telerehabilitation requires multi-faceted clinical reasoning when deciding what patient is appropriate for what types of telerehabilitation care. Safety considerations need to take into account physical and cognitive abilities of patients. Therapeutic and safety considerations were often made in consideration of family member availability, where they could be called upon to support and prepare for the therapeutic activities. Future studies are needed to understand more fully the range of needed safety considerations, best practices, and potential administrative policies when engaging in telerehabilitation, especially for patients with mental health conditions or significant cognitive decline. Future studies should also examine considerations around caregiver safety when supporting various telehealth-delivered rehabilitation activities. Questions remain around the extent to which providers should consider cognitive or physical screening protocols for determining an older caregiver's capacity for supporting a patient.

Telehealth technologies enhanced functionally tailored client-centered care and assessments by reaching into patients' home environment. Natural or contextualized functional activities carried out in the patient home, which includes task-oriented training, is consistent with evidence for improved carryover and increases in functional gains for patients undergoing neurorehabilitation (49). While telerehabilitation may not be appropriate in all patients, providers desired a hybrid system of using both telerehabilitation and in-person visits as a way to advance the rehabilitation care continuum. This finding is consistent with a recent study whereby physical therapy clinicians envision a hybrid approach to rehabilitation in going forward, even as pandemic-related needs for telehealth decrease (44).

The rapid and continued evolution of telerehabilitation services signal a critical need for clinical guidelines for conducting telerehabilitation. There remains a need for continued work in establishing both general guidelines for telerehabilitation assessment and intervention procedures (50, 51) and condition-specific telerehabilitation guidelines across the range of disabling conditions [e.g., (52)]. Efforts toward standardization of telerehabilitation care practices, clinical tools, and workflows are needed as telerehabilitation continues to mature (44, 53). Such efforts can improve rehabilitation practice and has the potential to improve the quality of patient care (52).

Study findings highlight the important role of leadership – at all levels - in facilitating telerehabilitation success. In the present case, this included support from local leadership (i.e., frontline clinic leaders who are responsible for executing day-to-day operations and implementing new initiatives, TR-EWI program directors, and facility leadership such as hospital directors), as well as administrative and programmatic support from regional (i.e., Veterans Integrated Services Network [VISN]) and national VHA leadership (e.g., Office of Rural Health, Office of Connected Care, VA Telehealth). The VHA invested early in telerehabilitation programs and research, such as the Low ADL Monitoring Program described by Bendixen and colleagues in 2007 (54), the Rural Veterans TeleRehabilitation Initiative launched in 2009 (20), and the 2017 national rollout of the TR-EWI program (16). These early and ongoing commitments by VHA leaders in promoting telerehabilitation established a strong foundation – one on which the VHA could quickly invest and support its robust transition during the pandemic. As the world continues to adapt during and beyond the COVID-19 pandemic, the VHA and its leadership continue to play an instrumental role in shaping telerehabilitation's maturation (45, 46).

Study findings are important for informing current and future VHA rehabilitation practice and initiatives. VHA research, clinical practice, and policy often intersect with public health concerns regarding individuals with disabilities (55, 56). Providing information to practicing clinicians regarding supports and strategies in the use of telehealth technologies can increase provider confidence, proficiency, and efficiency in enhancing and extending VHA rehabilitation services to those who may face rehabilitation access challenges.

Interpretation of findings presented are both enriched and limited by the nature of the qualitative approach. Findings contribute rich insight into a wide range of processes used by practicing VHA rehabilitation clinicians; they do not, however, provide insights into practice trends that may have transferability beyond our sample. Findings shed light on multiple factors within telerehabilitation practice that should be investigated in pursuit of optimizing patients' rehabilitation outcomes and providers' effectiveness and efficiency with telehealth technologies.

## Conclusion

The COVID-19 pandemic served as the catalyst for the rapid scaling up of telerehabilitation services across the country that in turn facilitated providers', health systems', and patients' readiness to participate in rehabilitation *via* telehealth. Providing telerehabilitation widely is an important long-term strategy for improving patients' access to a wide range of needed rehabilitation care. Clinical considerations are described for VHA rehabilitation providers to consider when using telehealth modalities in rehabilitation. VHA telerehabilitation has the potential to substantially transform rehabilitation care and maintain or improve rehabilitation outcomes, while enhancing both patients' care plan and the rehabilitation continuum.

## Data Availability Statement

The datasets presented in this article are not readily available because supporting data cannot be made openly available due to privacy requirements. Requests to access the datasets should be directed to Kimberly.findley@va.gov.

## Ethics Statement

The project was deemed a quality improvement project in accordance with the local and national legislation and institutional requirements. Ethical review, approval and written inform consent was not required.

## Author Contributions

CK, JH-G, KF, KM, and SR: study conception and design. CK, JH-G, KF: acquisition of data. CK, JK, SM, and MS: analysis of data. CK, JH-G, MS, KF, KM, and SR: interpretation of data. CK, JH-G, JK, SM, and MS drafted the initial work and all authors contributed critical appraisal and revision for important intellectual content. All authors contributed to the work and provide final approval of the version submitted for publication and agree to be accountable for all aspects of the work as presented.

## Funding

Funding was provided by the Office of Rural Health, U.S. Department of Veterans Affairs (OMAT-6145. Tele-rehabilitation Enterprise-Wide Initiative).

## Author Disclaimer

The opinions expressed herein are those of the authors and do not necessarily reflect those of the U.S. Department of Veterans Affairs or any of its affiliated institutions.

## Conflict of Interest

The authors declare that the research was conducted in the absence of any commercial or financial relationships that could be construed as a potential conflict of interest.

## Publisher's Note

All claims expressed in this article are solely those of the authors and do not necessarily represent those of their affiliated organizations, or those of the publisher, the editors and the reviewers. Any product that may be evaluated in this article, or claim that may be made by its manufacturer, is not guaranteed or endorsed by the publisher.
